# THE MORE, THE MERRIER? OLDER ADULTS’ LEISURE WITH OTHERS AND ASSOCIATIONS WITH WELL-BEING OVER TIME

**DOI:** 10.1093/geroni/igae098.2436

**Published:** 2024-12-31

**Authors:** Amy Rauer, Meagan Stewart, Katherine Fiori, Christina Marini, Jeremy Kanter

**Affiliations:** University of Tennessee, Knoxville, Tennessee, United States; University of Tennessee, Knoxville, Tennessee, United States; Adelphi University, Garden City, New York, United States; Adelphi University, Garden City, New York, United States; University of Tennessee, Knoxville, Tennessee, United States

## Abstract

The benefits of leisure for older adults are well-documented, as is the key role that social networks play in facilitating more opportunities for leisure. However, it is unclear if these opportunities are uniformly beneficial over time or if engaging in leisure with certain social ties (or alone) are more advantageous, particularly when barriers to social activities are greater (i.e., COVID-19 pandemic). Thus, we examined older adults’ leisure alone and with others and its links to well-being using a sample of 136 older adults in the U.S. (Mage = 68, range: 50-91; 69% females; 93% White; 72% partnered; 61% retired). Participants reported on leisure at three time points (Summer 2021/Fall 2021/Winter 2022) using a modified version of the Pittsburgh Enjoyable Activities Scale (Pressman et al., 2009). Older adults were most likely to do activities with romantic partners, followed by activities alone, with leisure with friends and family least likely. Although more leisure engagement overall was associated with better well-being (depressive symptoms, anxiety symptoms, loneliness, life satisfaction, perceived mattering), it was primarily leisure with others that appeared beneficial (romantic partner, friends, family). In fact, greater solitary leisure was linked to feeling less important (perceived mattering). Given that leisure with a romantic partner and with family declined when COVID-19 cases peaked (in contrast to the stability of overall leisure, solitary leisure, and leisure with friends), it appears that older adults were still able to do things they enjoyed, but the limitations on with whom they could engage in leisure were not without consequence.

